# Of Mice and Culture: How Beliefs About Knowing Affect Habits of Thinking

**DOI:** 10.3389/fpsyg.2022.917649

**Published:** 2022-07-12

**Authors:** Hiroaki Morio, Saiwing Yeung, Kaiping Peng, Susumu Yamaguchi

**Affiliations:** ^1^Faculty of Informatics, Kansai University, Osaka, Japan; ^2^Zillow Group, Seattle, CA, United States; ^3^Department of Psychology, Tsinghua University, Beijing, China; ^4^Department of Social Psychology, The University of Tokyo, Tokyo, Japan

**Keywords:** naïve dialecticism, cultural differences, attitude structure, ambivalence, moment-to-moment evaluations

## Abstract

Recent research suggests that individuals from East Asian and Western cultures differ in the degree to which they hold a folk world view known as naïve dialecticism, which is characterized by tolerance for contradiction, expectation of change, and cognitive holism. The current research utilizes the Mouse Paradigm to investigate the dynamic nature of naïve dialecticism in real time by measuring individuals’ fluctuations in judgment during the process of contemplation. The results showed cultural differences in dynamic measures of evaluation process: Japanese participants took more time to stabilize their thought and showed more fluctuations in their judgment than American participants. These cultural differences were fully mediated by individual differences in levels of naïve dialecticism as measured by the level of dialectical self-views. Implications for cultural psychology and the psychology of dialectical thinking are discussed.

## Introduction

Studies have revealed significant cultural differences in cognition between individuals of Western and East Asian cultural descent (Nisbett et al., 2001; Spencer-Rodgers et al., 2018a). Nisbett et al. (2001) proposed a classification in terms of “analytic” versus “holistic” thought to explain those cognitive differences. In contrast to Western analytic cognition, which focuses on objects, categories, and formal logic, they argue that East Asian holistic cognition focuses on the environment and relies less on categories and formal logic. More specifically, Peng and Nisbett (1999) argue that East Asians’ holistic thought is characterized by dialectical reasoning and judgments. Originating from Taoist, Confucian, and Buddhist traditions, *naïve dialecticism* refers to an East-Asian lay belief system in which understanding may be achieved through the acceptance of change, contradiction, and relations among parts of the whole (Peng et al., 2001). This perspective argues that cultural differences in psychological processes and behavior may be attributed to differences in the naïve epistemologies, or beliefs about knowing and learning, endorsed by individuals from East Asian and Western cultures.

One implication of holding a dialectical world view is that, in line with a greater tolerance for contradiction, dialectical thinkers might simultaneously hold inconsistent or contradictory viewpoints. For this reason, dialectical thinking is assumed to be associated with longer-term and more fluctuating processes of contemplation. Indeed, East Asians are more likely to accept seeming contradictions without need for resolution than are Westerners (see Spencer-Rodgers et al., 2018a, for a review).

In the present study, we assumed that individuals in dialectical cultures—and individuals who are high in dispositional dialecticism—would be more comfortable seeking and accepting the coexistence of opposing attitudes and emotions. As a result, their dialectical tendency may lead to more calibration and change in their thought processes, which in turn would lead to a longer evaluation process. We examined this hypothesis in the current study using an experimental procedure that is well suited to studying the dynamism in the process of evaluation.

### Measurement of Evaluation Process

Most studies of human reasoning and judgment focus their investigation on the final outcome or result of a mental process. For example, the Likert scale, one of the most popular measurement methods, captures responses at the end of a cognitive or evaluative process. While such responses can capture the result of the evaluation that produced the responses, the scale cannot tap into the process through which an individual reached the final conclusion. An understanding of such processes may be particularly germane to the study of individuals who differ in their beliefs about knowing. For example, in deciding their opinion of a particular topic, one individual may generate one mildly positive argument toward the topic and thus decide to give a mildly positive evaluation. On the other hand, another person (perhaps especially one who tends to think dialectically) might evaluate many arguments, both for and against the subject matter, and finally decide to give a mildly positive evaluation. These two individuals would show identical ultimate responses as a result of very different contemplative processes.

To overcome this problem, Vallacher et al. (1994) developed a computer-based technique known as the Mouse Paradigm, which allows for the continuous measurement of judgment during an evaluation process and thus enables us to analyze the whole process of one’s evaluation. The Mouse Paradigm is designed so that respondents have to provide spontaneous, unplanned narratives for a given topic. As a result, their narratives are not well-prepared formal speeches, and they reflect the dynamic mental processes of evaluation. This technique has been used to measure dynamic properties of attitudes (Pryor et al., 2004), social judgment (Vallacher et al., 1994), and self-evaluation (Vallacher et al., 2002; Morio et al., 2007).

In the Mouse Paradigm, participants are asked to sit alone and talk freely and spontaneously about a given topic for a set period of time, as the narrative is recorded. The recorded narrative is then played back to the participants. While listening to their own voice, the participants indicate their feeling toward the topic at the moment of recording, using a mouse cursor on a computer screen. The participants are instructed to move the cursor closer to the center of the screen if they feel positive about the topic, and further away if negative. The position of the mouse cursor is recorded every 100 ms, producing online time-series data for the individual’s evaluation of the target topic. The frequency and nature of these judgments provides quantitative, dynamic data with enough resolution for researchers to examine the dynamic properties of the evaluation.

In the present study, two indices that reflect different dynamic properties of evaluation processes are calculated from mouse trajectories: duration of contemplation and number of fluctuations. These two indices were expected to yield higher score as participants’ thinking becomes more dialectical. The first index, duration of contemplation, represents the amount of time it takes for the evaluation of a target topic to stabilize until the subject reaches a final rating of the target’s favorability. The second index is the frequency of fluctuation, which indicates how often a participant’s evaluation changed direction before a final decision was reached. The more often a person’s evaluation changes direction, either from positive to negative or negative to positive, the higher the index. These two indices represent the uncertainty and volatility of the mental process and should reflect cultural differences in naïve epistemologies. Specifically, we hypothesized that Japanese participants would spend a longer time contemplating their decision, and would show more fluctuation in their evaluative processes, compared to American participants.

## Materials and Methods

### Participants

The Japanese participants (*N* = 59) were students at the University of Tokyo, who ranged in age from 18 to 26 (*M* = 20.9). Twenty-four percent of the sample was female. The Japanese participants were given 500 yen in exchange for their participation.

The American participants (*N* = 68) were students at the University of California, Berkeley. Gender and age were unfortunately not assessed in this sample. However, it should be expected that the American participants would be of similar ages to the Japanese participants but with a higher female/male ratio. A study conducted with the same subject pool as the American sample at the time had an average age of 22. The American participants were given partial credit for a psychology course in exchange for their participation.

The sample sizes were determined by the resource constraints. *Post hoc* sensitivity analysis with the current sample size, alpha of 0.05, and power = 0.95 yielded critical *η^2^* of 0.095.

### Overview of Procedure

Participants were tested individually as a part of a larger study. After having completed an informed consent form, participants were placed alone in a small room with a personal computer running Windows XP with an LCD monitor. Prior studies using the Mouse Paradigm have confirmed that being alone encourages participants to express their thoughts more freely. The participant was instructed to complete the Mouse Paradigm (described below), followed by a series of tasks unrelated to the present study. After the filler tasks, the participant was asked to complete a questionnaire containing the Dialectical Self Scale (described below). Finally the participant was debriefed and dismissed.

### Mouse Paradigm

The software for the Mouse Paradigm was developed by the authors with Microsoft Visual Basic 6.0. Timer object was used to call GetCursorPos API to record the mouse cursor position every 100 ms. To begin the Mouse Paradigm, the experimenter helped the participant put on a headset with a built-in microphone, which was used to record the participant’s verbalized thoughts, and later to replay them. During the initial recording phase, the first of two topics was displayed on the screen, either (a) the importance of recycling or (b) homosexuality. These topics were selected because of their controversial nature among college students in both cultures. The order of topics was randomized in both samples. Participants were instructed to talk freely for 1 min about how they felt about the topic.

**Figure 1:**
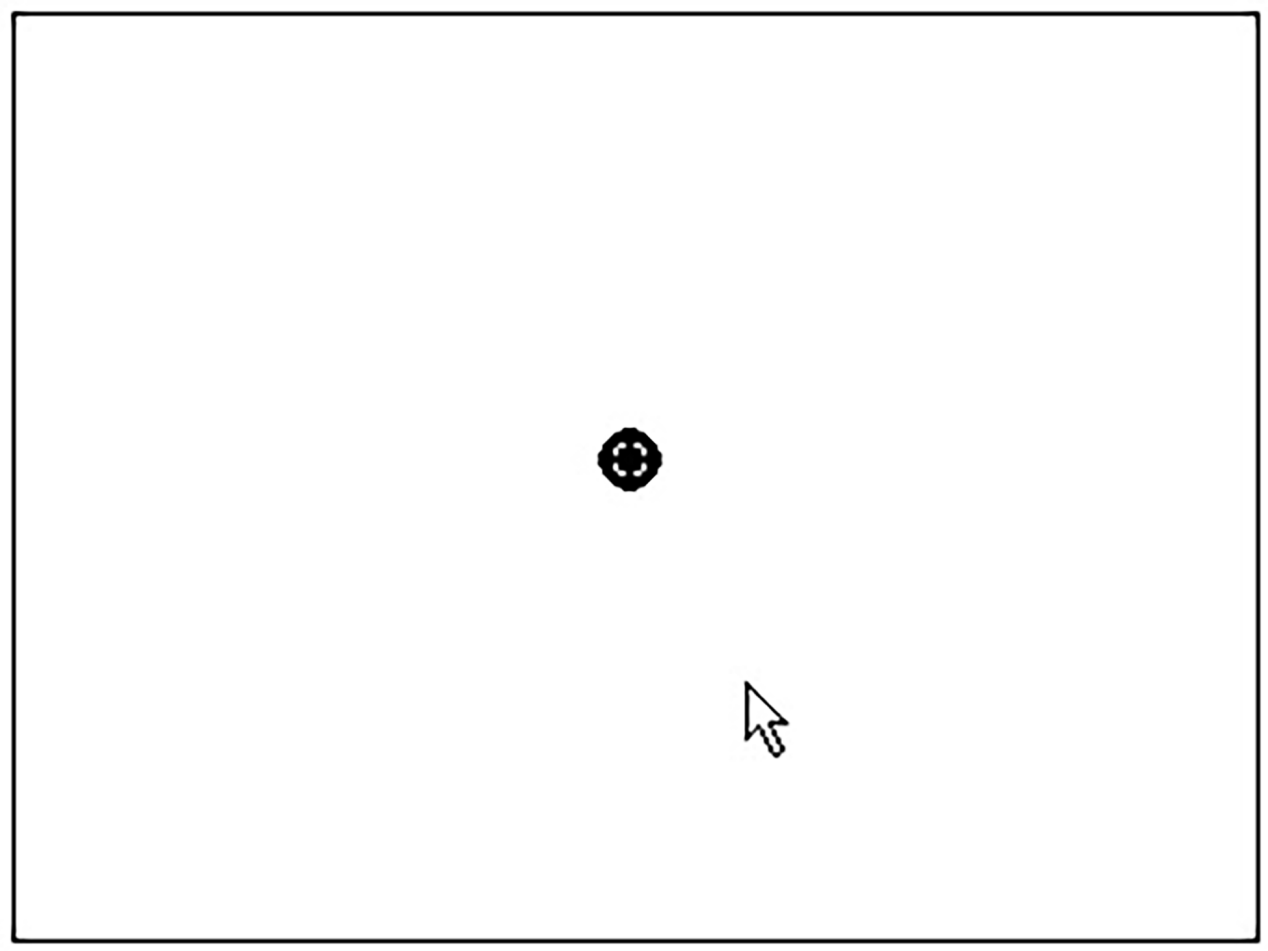
A schematic of the computer screen during the rating phase.

Next, during the rating phase, the computer displayed a blank screen containing two objects, the mouse cursor and the target (see Figure 1). The computer replayed the recording of the participant’s narrative. Participants were instructed to recall their moment-to-moment feelings toward the topic throughout the recording phase, and indicate the valence using the mouse cursor. The more positively they felt toward the topic, the closer to the target they should move the cursor. The more negatively they felt toward the topic, the farther away they should move the cursor. The entire recording was played back and the participants were asked to move the cursor accordingly and continuously until the end of the rating phase. After the rating phase was over, the program moved to the second of the two topics.

### Dialectical Self Scale

In addition to the Mouse Paradigm, participants also completed the Dialectical Self Scale (DSS), a paper-and-pencil measure of naïve dialecticism in the domain of self-perception (Spencer-Rodgers et al., 2018a,b). The DSS has been applied to research in various areas including the self (Spencer-Rodgers et al., 2004; Chen et al., 2006, 2013), personality (Fetvadjiev et al., 2018a,b; Na et al., 2020), emotions (Sims et al., 2015; Hideg and Kleef, 2017; Lu et al., 2017b), intergroup conflicts (Lu et al., 2017a, 2020), resilience capacity (Zheng et al., 2020), attitudes toward social issues (Hideg and Ferris, 2017; Lee et al., 2020; Li et al., 2020), employee performance (Bai et al., 2015), and causal attributions (Li et al., 2016). The original version in English was translated into Japanese *via* back translation (Brislin, 1980). Participants rated the 32 items in the scale using a 7-point Likert scale (1 = “strongly disagree” and 7 = “strongly agree”). Previous research has not reported any consistent age or gender differences on DSS. The scale consists of three subscales: Tolerance for Contradiction, Cognitive Change, and Behavioral Change. The Tolerance for Contradiction subscale measures the degree to which the person is comfortable with contradicting views about the self. The Cognitive Change subscale measures how likely it is that a person’s overall self-beliefs or attitudes will change over a period of time or at the introduction of new information. The Behavioral Change subscale assesses the likelihood that a person will behave differently under different situations. Zell et al. (2013) reported that the reliability of the DSS ranged from 0.65 to 0.84 among young adults across 19 nations. The three subscales also have good internal consistency among American samples (Martín-Fernández et al., 2022). The DSS has demonstrated moderate convergent validity (Spencer-Rodgers et al., 2004).

By using the individual measure of naïve dialecticism, we attempted to provide evidence that the predicted cultural difference in the Mouse Paradigm reflects an underlying individual difference. In particular, we focused on the 13-item Tolerance for Contradiction subscale, predicting that participants with higher Tolerance for Contradiction scores would contemplate longer and fluctuate more in their evaluation process. Moreover, it as hypothesized that individual differences in Tolerance for Contradiction would mediate the predicted cultural differences in mental processes.

## Results

### Preparation of Dependent Variables

**Figure 2:**
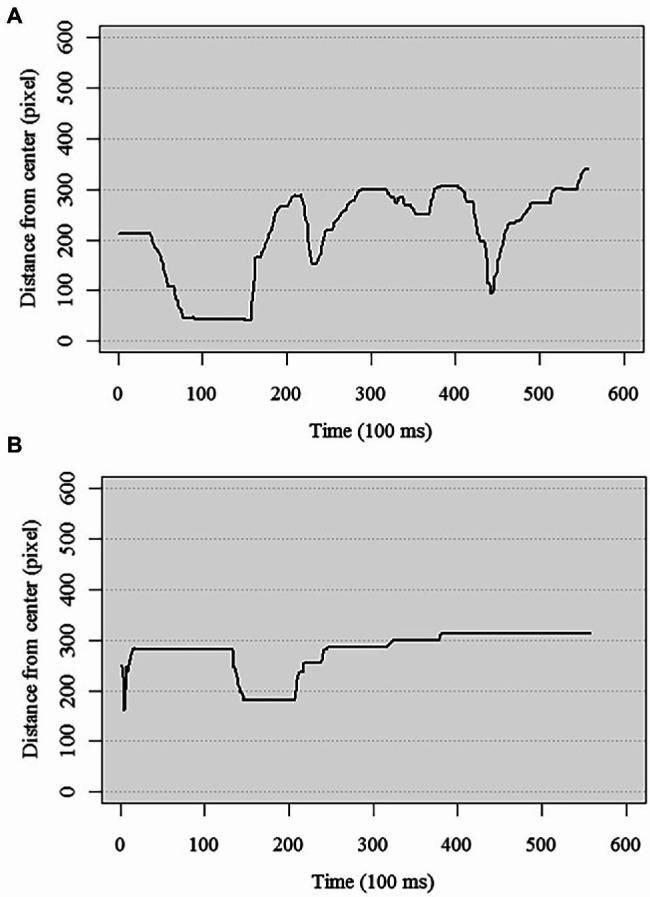
Sample distance over time plots of an American participant with high **(A)** and low **(B)** Contradiction subscale score.

To prepare the two dependent variables, the duration of contemplation and the degree of fluctuation, the coordinates of the mouse cursor as captured every 100 ms were first converted to their distance from the screen center. Figure 2 shows sample plots of the trajectory of distances over time by two different participants.

For the duration index, the amount of time for the trajectory to stop changing the distance from the center was computed with 100 ms as the unit of time. If the evaluation does not change at all from the beginning, the value is 0, and if the evaluation does not stabilize during the duration of recoding the 60 s, the value is 600. It was then subtracted from a theoretical maximum of 600. Because the duration index is reverse scored, a larger score indicates shorter duration.

The second index, fluctuation, reflects the number of times the trajectory changed its direction, either from positive to negative or from negative to positive. A larger fluctuation index indicates more fluctuations in the direction of evaluations during the rating phase. Both of the dependent variables were totaled across the two topics and then log-transformed with a base of 10 because of their skewed distributions (for the raw duration index, skewness was 1.45 and kurtosis was 1.22; for the raw fluctuation index, skewness was 1.22 and kurtosis was 1.92). Means and standard deviations for the two variables in each cultural group are presented in Table 1.

**Table 1:** Means and standard deviations of Tolerance for Contradiction score and mouse paradigm indices by culture.

	United States		Japan	
	*M*	*SD*	*M*	*SD*
Tolerance for Contradiction score^**^	55.21	10.14	61.24	7.79
Mouse Paradigm indices				
Duration index^*^	2.12	0.35	1.97	0.33
Fluctuation index^*^	1.68	0.45	1.84	0.28

The duration index of the Mouse Paradigm is reverse scored. Thus, a higher score indicates a shorter duration before stabilization.

* *p* < 0.05;

** *p* < 0.01.

### Cultural Differences

As can be seen in Table 1, the Japanese participants took more time to stabilize their evaluations of the two topics, and showed more fluctuations in their evaluations, compared to American participants. These cultural differences are consistent with our hypotheses. ANOVAs revealed that culture had a significant effect on duration, *F*(1, 125) = 5.28, *p* = 0.03, *η^2^* = 0.041, and on fluctuation, *F*(1, 125) = 5.59, *p* = 0.02, *η^2^* = 0.043.^1^

Japanese participants also had higher scores on the Tolerance of Contradiction subscale of the DSS than did their American counterparts, *F*(1, 125) = 13.8, *p* = 0.001, *η^2^* = 0.099. It should be noted here, however, that the internal reliability (Cronbach’s *α*) of the subscale was low for Japanese participants (*α* = 0.50), although it was satisfactory for the American participants (*α* = 0.75). The low reliability of the scale in the Japanese sample necessitates caution in interpreting results concerning this subscale.

### Mediation Analyses

We hypothesized that tolerance for contradiction, a component of dialectical thinking, would mediated cultural differences in dynamic thought processes as measured by the Mouse Paradigm. As an illustration, Figure 2 presents the trajectories of two American participants who are representative of those high or low in tolerance for contradiction. The figures show how their evaluation of the target changed over the 60s rating period: how long it took for them to stabilize the evaluation, and how much their thought process fluctuated.

Toward this end, we carried out a mediation analysis following the procedure outlined by Baron and Kenny (1986). As we have shown in the previous section, the first two criteria in their procedure have been established; namely: (a) a significant effect of the independent variable (i.e., culture) on the dependent variable (the duration index and the fluctuation index) and (b) a significant effect of the independent variable (culture) on the proposed mediator (tolerance for contradiction). The third requirement of the mediation analysis (Step 3) is a significant correlation between the proposed mediator (tolerance for contradiction) and the dependent variable (the duration and fluctuation indices) after controlling for the independent variable (culture). To examine the third criterion, we took the partial correlation between Tolerance for Contradiction and the two indices of dynamism with the effect of culture being controlled. The results indicated that the third requirement is met for both the duration and fluctuation indices, *r* = −0.20, *p* = 0.03 and *r* = 0.19, *p* = 0.04, respectively. These results indicate that those with higher Tolerance for Contradiction took longer time of contemplation and showed more fluctuations and change in their evaluation process. Finally, we tested the fourth criterion that the effect of the independent variable (culture) on the dependent variable (duration and fluctuation indices) drops to non-significance when the mediator (tolerance for contradiction) is controlled for. An analysis of covariance (ANCOVA) with Tolerance for Contradiction score being a covariate was conducted for each of the two indices. As required, the ANCOVAs did not yield a significant effect of Culture for the duration index and the fluctuation index [*F*(1, 124) = 2.30, *p* = 0.13, *η^2^* = 0.017 and *F*(1, 124) = 2.53, *p* = 0.11, *η^2^* = 0.020, respectively]. In addition, the effect of Tolerance for Contradiction was significant for both the duration index and the fluctuation index as required for a demonstration of mediation [*F*(1, 124) = 4.92, *p* = 0.03, *η^2^* = 0.039 and *F*(1, 124) = 4.75, *p* = 0.03, *η^2^* = 0.037, respectively]. Finally, Sobel’s test was conducted using the bootstrapping procedure (Baron and Kenny, 1986; Shrout and Bolger, 2002; Preacher and Hayes, 2004). The results of the test were significant at alpha <0.05 for both of the two dynamism indices. The 95% confidence intervals are *Lower limit of CI =* −0.0963 and *Upper limit of CI =* −0.0036 for the duration index and *Lower limit of CI =* 0.0097 and *Upper limit of CI =* 0.0921 for the fluctuation index. These results showed that the cultural difference in the duration of contemplation and number of fluctuation are both fully mediated by individuals’ degree of tolerance for contradiction.

## Discussion

The effects of naïve dialecticism on cognition and behavior have been previously noted (Peng and Nisbett, 1999), but the online process of evaluation has not been examined. This study demonstrated that the consequences of dialectical thinking are not limited to final result of an evaluation. Instead, consistent with our hypotheses, participants from a dialectical culture (Japan) took more time before stabilizing their evaluations of a social topic, and showed more fluctuation in their evaluations, relative participants from a non-dialectical culture (the United States). To the best of our knowledge, this is the first study to report such differences.

We also found that members of the two cultures differed in the degree to which they endorsed a self-view in which contradiction is tolerated, as measured by the Tolerance for Contradiction subscale of the DSS, and, further, that participants’ Tolerance for Contradiction scores affected the duration and fluctuation of their evaluation of social topics. More importantly, the present study demonstrated that Tolerance for Contradiction mediated cultural differences in the duration index (how long it takes to stabilize one’s evaluation) and the fluctuation index (how frequently one’s evaluation fluctuates before stabilization). This mediating effect by tolerance for contradiction for the indices of dynamism provides further evidence supporting our claim that cultural difference obtained for the dynamic measures of evaluation process reflects levels of naïve dialecticism. However, it should be remembered that the internal consistency of the Tolerance for Contradiction scale was low among Japanese participants, a limitation that should be addressed through replication. This finding may reflect subtle translation differences in the scale, or the possibility that scale items might need modifications to more adequately tap individual differences in dialecticism among Japanese. While Zell et al. (2013) reported a good internal consistency of the global DSS with the 32 items in various countries, reliability and validity of the Tolerance for Contradiction scale has not been examined in Japan. More thorough cross-cultural research focusing on the subscale is needed. It should be also noted that the accuracy of the measurement made by the software used in this study was not validated, thus limiting the validity of the measurement. The present research suggests that an evaluation process can be influenced by both cultural- and individual-level tolerance for contradiction, which is a part of naïve dialecticism. The observed cultural difference in duration of mental contemplation was fully accounted for by the individual-level naïve dialecticism. On the other hand, the individual-level naïve dialecticism could not explain the cultural difference in fluctuations during the evaluations. This inconsistency indicates that a concurrent examination of the dynamism in evaluation processes at the two different levels (i.e., culture and individual) is important. We look forward to future studies that will shed light on the differential influences of naïve dialecticism at the cultural and individual levels.

Regardless of such limitation, the highly dynamic evaluation process found in this study, especially for individuals with high dialecticism (culturally or individually), suggests that studies on evaluation or dialectical thinking can benefit from close attention to the dynamism of evaluation processes. Traditional psychological measurements might work well for cultures or individuals where linear thinking is the norm, but for dialectical thinkers, more sophisticated techniques such as the Mouse Paradigm is required to reveal differences that cannot be unearthed otherwise.

Capturing the dynamism of moment-to-moment evaluation process as a measure of dialectical thinking would be quite useful in elucidating the nature of the evaluation process. The results in this converge with Zhang et al. (2015) which used endorsing two opposing opinions at the same time as a measure of dialectical thinking and found a relationship between the DSS score and the dialectical thinking measure. Further study is needed to examine the relationship between the dynamism of evaluation and Zhang et al.’s (2015) static measure of dialectical thinking. Measuring the characteristics of evaluation process is also expected to be useful in distinguishing similar individual differences in cognitive process, such as indecisiveness (Ng and Hynie, 2014, 2016; Li et al., 2016).

## Data Availability Statement

The raw data supporting the conclusions of this article will be made available by the authors, without undue reservation.

## Ethics Statement

The studies involving human participants were reviewed and approved by The University of Tokyo, Department of Social Psychology ethics committee. The patients/participants provided their written informed consent to participate in this study.

## Author Contributions

HM and KP conceptualized and designed the studies. HM and SaY prepared, translated, and refined the materials. HM, SaY, and SuY collected, analyzed, and interpreted the data. KP drafted the article. All authors contributed to the article and approved the submitted version.

## Funding

Part of this research was supported by grants to HM and SuY from the Japan Society for the Promotion of Science (16730305, 16530397, and 19K03203).

## Conflict of Interest

SaY was employed by the company Zillow Group.

The remaining authors declare that the research was conducted in the absence of any commercial or financial relationships that could be construed as a potential conflict of interest.

## Publisher’s Note

All claims expressed in this article are solely those of the authors and do not necessarily represent those of their affiliated organizations, or those of the publisher, the editors and the reviewers. Any product that may be evaluated in this article, or claim that may be made by its manufacturer, is not guaranteed or endorsed by the publisher.

## Footnotes

^1^Nonparametric test was used to examine if the effect of culture was significant on the dependent variables before log-transformation. Median duration index before conversion for American and Japanese samples were 103.5 and 66; the distributions in the two groups differed significantly (Mann–Whitney U = 1,523, *p* = 0.02). Median fluctuation index before conversion for American and Japanese samples were 58 and 80; the distributions in the two groups differed significantly (Mann–Whitney U = 2,430, *p* = 0.04).
